# Mental Health in Elite Student Athletes: Exploring the Link Between Training Volume and Mental Health Problems in Norwegian College and University Students

**DOI:** 10.3389/fspor.2022.817757

**Published:** 2022-02-01

**Authors:** Michael Grasdalsmoen, Benjamin Clarsen, Børge Sivertsen

**Affiliations:** ^1^Department of Sport, Food and Natural Sciences, Western Norway University of Applied Sciences, Bergen, Norway; ^2^Sports Trauma Research Center, Department of Sports Medicine, Norwegian School of Sport Sciences, Oslo, Norway; ^3^Department of Disease Burden, Norwegian Institute of Public Health, Bergen, Norway; ^4^Department of Health Promotion, Norwegian Institute of Public Health, Bergen, Norway; ^5^Department of Research & Innovation, Helse Fonna HF, Haugesund, Norway; ^6^Department of Mental Health, Norwegian University of Science and Technology, Trondheim, Norway

**Keywords:** elite athlete, mental health-related quality of life, epidemiology-descriptive, college & university students, self-harm behavior

## Abstract

**Objectives:**

To examine mental health problems among elite athletes in a student population, compared to the general student population, and to explore the association between weekly hours of training across mental health indicators.

**Methods:**

Data are from a national study from 2018 of all college and university students in Norway. Participants indicated if they considered themselves to be an elite athlete, and how many hours per week they trained. Mental health problems were assessed using several well-validated questionnaires.

**Results:**

Among 50,054 students, 1.3% identified themselves as elite athletes. Both male and female elite athletes had generally better mental health across most health outcomes, reporting fewer mental health problems, less loneliness, higher satisfaction with life, more positive affect, and fewer alcohol problems. Elite athletes in team sports had slightly better mental health compared to athletes of individual sports. Increased hours of weekly exercise was associated with better mental health. However, there was generally little to be gained from increasing the amount of training from 7–10 hours/week to 14+ hours per week. Female athletes who trained 14 or more hours per week reported poorer mental health across most outcome measures.

**Conclusion:**

This study showed that both male and female elite athletes generally had better mental health across a range of health outcomes, when compared to the general student population. The study also found a positive dose-response relationship between weekly hours of training and mental health, but also a worsening of mental health for females at the extreme end of exercise continuum. The self-report nature of this student sample means that care should be taken when generalizing to other studies of elite athletes.

## Introduction

Physical exercise has unquestionable benefits for health, and taking part in regular exercise has been shown to prevent a host of non-communicable diseases (NCDs), including cardiovascular disease and type 2 diabetes (Lee et al., 2012). But despite overwhelming evidence of the many health benefits of physical exercise, it has been suggested that there might be a curvilinear relationship between the amount of physical exercise and exercise-induced improvements of somatic health (Mons et al., 2014; Williams and Thompson, 2014; Schnohr et al., 2015). Although such findings may lead to the speculation that physical exercise may be harmful at a certain dose, there is currently no known upper limit in terms of the somatic health benefits of physical exercise in healthy individuals (Eijsvogels and Thompson, 2015).

There is similar evidence showing that physical exercise also has large positive effects on mental health, especially in the case of depression (Kvam et al., 2016; Gordon et al., 2018). However, the research literature regarding the nature of this relationship also remains equivocal. While a large US study of 1.2 million individuals found a strong link between physical exercise and improved mental health (Chekroud et al., 2018), they also concluded that more exercise was not always better. Exercising 23 or more times per month, or longer than 90 min per session, was associated with worse mental health. In contrast, a recent national study of all college and university students in Norway found that the more physical exercise, the better; both in terms of exercise frequency and duration (Grasdalsmoen et al., 2020a). However, none of these studies focused specifically on individuals at the extreme end of the exercise spectrum, elite athletes.

Despite the utmost importance of optimal physical and mental health when performing at a top international level of any sport, there are relatively few studies investigating in detail the mental health of elite athletes, and findings remain inconclusive (Reardon and Factor, 2010; Reardon et al., 2019). While some studies have reported prevalence rates of mental disorders and substance use disorder to be higher or comparable to the general population (Schaal et al., 2011; Rice et al., 2016; Gouttebarge et al., 2019; Akesdotter et al., 2020; Purcell et al., 2020), other studies have found lower suicide rates when compared to the general population (Maron et al., 2014; Rao et al., 2015; Lehman et al., 2016). Similarly, in one of the largest studies in this field, a recent study of US Olympians found a lower risk of mental health problems and suicide for this group, compared to the general population (Duncombe et al., 2020). In a consensus statement from IOC, it was concluded than more studies with large reference groups were needed to address specific domains of mental health outcomes in elite athletes, and also to examine whether there may by gender-specific patterns in the associations between the amount of exercise and mental health indicators (Reardon et al., 2019).

Based on these considerations, the aim of the current study was twofold; first, to investigate in detail the prevalence of mental health problems across a range of outcome measures in elite athletes compared to the general student population, and second, to explore the linear vs. curvilinear association between weekly hours of training across all mental health indicators.

## Methods

### Procedure

The current paper used data from the SHoT2018 study (*Students' Health and Wellbeing Study)*, a large national survey of students enrolled in higher education in Norway. The SHoT2018 is a comprehensive survey of several domains of mental health and lifestyle factors, distributed electronically through a web-based platform at the University of Oslo. Details of the study has been published elsewhere (Sivertsen et al., 2019a), but in short, SHoT2018 was conducted between February 6 and April 5, 2018, and invited all full-time Norwegian students pursuing higher education, both in Norway and abroad. In all, 162,512 students fulfilled the inclusion criteria, of whom 50,054 students completed the online questionnaires (after being sent two reminders), yielding a response rate of 30.8%.

### Elite Athlete and Exercise

The students were first presented with the following brief definition of physical exercise: “*With physical exercise, we mean that you, for example, go for a walk, go skiing, swim or take part in a sport*.” Physical exercise was then assessed using three sets of questions, assessing the average number of times exercising each week, and the average intensity and average hours each time (Kurtze et al., 2007): (1) “*On an average week, how frequently do you perform physical exercise?”* (Never, Less than once a week, Once a week, 2–3 times per week, Almost every day); (2) “*If you perform physical exercise as frequently as once or more times a week: How hard do you push yourself?”* (I take it easy without breaking into a sweat or losing my breath, I push myself so hard that I lose my breath and break into a sweat, I push myself to near-exhaustion); and (3) “*How long does each session last?”* (Less than 15 min, 15–29 min, 30 min to 1 h, More than 1 h.”) This 3-item questionnaire has previously been used in the large population-based Nord-Trøndelag Health Study (HUNT) (Kurtze et al., 2007, 2008). Detailed information on the physical exercise items in the SHoT2018 study has been published elsewhere (Grasdalsmoen et al., 2019, 2020a,b).

If respondents answered that they exercised “almost every day” on the frequency item, they were then asked if they considered themselves to be an “*elite athlete”* (yes/no), and if so, how many hours per week they trained (drop-down menu: 0 to 40 h). For the item of weekly hours of training, we categorized all responses into “0–2 h/wk, 3–6 h/wk, 7–10 h/wk, 11–13 h/wk, and 14+ h/wk (based on the distribution of the responses). Due to restrictions related to statistical power, we were unable to further explore in detail those training more than 14 h/wk. Finally, those self-categorized as an elite athlete were also asked (in free text) which sport they considered themselves as an elite athlete. For purposes of the present study, all responses were manually coded as either *individual* or *team* sport.

### Mental Health Problems

Mental health problems were assessed by the widely used Hopkins Symptoms Checklist (HSCL-25) (Derogatis et al., 1974), derived from the 90-itemSymptom Checklist (SCL-90), a screening tool designed to detect symptoms of anxiety and depression. Several factor structures and cut-offs for clinical levels have been proposed for the HSCL-25 (Ventevogel et al., 2007; Glaesmer et al., 2014). An investigation of the factor structure based on the SHoT2014 dataset showed that a unidimensional model had the best psychometric properties in the student population and not the original subscales of anxiety and depression (Skogen et al., 2017). We have chosen to follow this recommendation in the present study. As recommended in previous publications (Derogatis et al., 1974), the average scores on the HSCL-25 of ≥ 1.75 and <2.00, and >2.00, were used as cut-off values for identifying moderate and high levels of mental health problems, respectively. Details on development of mental health problems in the SHoT waves were recently published by Knapstad et al. (2019).

### Mental Disorders

Self-reported mental disorders were assessed by a pre-defined list adapted to fit this age-cohort. The list was based on a similar operationalisation used in previous large population-based studies [the HUNT study (Krokstad et al., 2013)] and included several subcategories for most conditions/disorders (not listed here). For mental disorders, the list comprised the following specific disorders/group of disorders: attention-deficit/hyperactivity disorder (ADHD), anxiety disorder, autism/Asperger, bipolar disorder, depression, posttraumatic stress disorder (PTSD), schizophrenia, personality disorder, eating disorder, Tourette's syndrome, obsessive-compulsive disorder (OCD), and others. The list contained no definition of the included disorders/conditions. Due to statistical power limitations, we only included anxiety and depressive disorders, in addition to eating disorders.

### Self-Harm and Suicidal Behavior

History of non-suicidal self-harm (NSSH) and suicide attempts were assessed with two items drawn from the Adult Psychiatric Morbidity Survey (APMS) (McManus et al., 2016); “*Have you ever made an attempt to take your life, by taking an overdose of tablets or in some other way?”*, and “*Have you ever deliberately harmed yourself in any way but not with the intention of killing yourself? (i.e., self-harm)”* If respondents answered yes to any item, the timing of the most recent episode was assessed, using the following response options: “last week”, “past year”, “more than a year ago, but after I started studying at the university”, and “before I started studying at university”. For purposes of the current study, we created a joint variable encompassing students that reported positive on any of these four items, and if they indicated the most recent episode to be *after* they started studying at university. More detailed information about self-harm and suicidal behavior in SHoT2018 has been published elsewhere (Sivertsen et al., 2019b).

### Life Satisfaction

The Satisfaction With Life Scale (SWLS) (Diener et al., 1985) is a 5-item scale designed to measure global cognitive judgments of one's life satisfaction (not a measure of either positive or negative affect). In the current study, the SWLS was analyzed in three ways: (1) as a continuous total score (range 5–35), (2) using pre-defined categories (*dissatisfied*: total SWLS score 5–19; *neutral*: total SWLS score 20–25, and *satisfied*: total SWLS score 26–35); and (3) dichotomously, using a total SWLS total score of <19 as the cut-off value indicating poor life satisfaction. The Cronbach's alpha of the SWLS in the current study was 0.89.

### Loneliness

Loneliness was assessed using an abbreviated version of the widely used UCLA Loneliness Scale, “The Three-Item Loneliness Scale (T-ILS)” (Hughes et al., 2004). The T-ILS has displayed satisfactory reliability and both concurrent and discriminant validity in two US nationally representative population-based studies (Hughes et al., 2004), and also performed well among US college students (Matthews-Ewald and Zullig, 2013). The three items were analyzed separately, and each item was dichotomized using “often” or “very often” as cut-off value. The Cronbach's alpha of the T-ILS in the current study was 0.88.

### Perfectionism

Perfectionism was assessed by the short version of the Perfectionism subscale from the Eating Disorder Inventory (EDI) (Garner et al., 1985). The Perfectionism subscale (EDI-P) comprises two dimensions: socially prescribed perfectionism and self-oriented perfectionism, and this two-factor model has been supported in both clinical (Lampard et al., 2012) and non-clinical (Muro-Sans et al., 2006) adolescent samples. The Cronbach's alpha of the EDI in the current study was 0.81.

### Disturbed Eating Patterns

Disturbed eating patterns were assessed by the Eating Disturbance Scale (EDS-5) (Rosenvinge et al., 2001), a brief screening instrument for problematic eating in normal populations. The EDS-5 has been shown to have good concurrent and construct validity, and a sensitivity and specificity of 0.90 and 0.88 with respect to DSM-IV eating disorders (Rosenvinge et al., 2001). The Cronbach's alpha of the EDS-5 in the current study was 0.83.

### Positive Affect

The Positive and Negative Affect Schedule (PANAS) is a 20-item questionnaire which comprises two subscales, one that measures positive affect (positive affect) and the other which measures negative affect (NA). A sum score is calculated with higher scores representing greater positive affect. The Cronbach's alpha for the positive affect subscale in the current study was 0.91. The NA subscale was not included in the SHoT study.

### Sleep Duration and Insomnia

Participants' self-reported usual bedtime and bed-rise time were indicated in hours and minutes, and data were reported separately for weekdays and weekends. Time in bed (TIB) was calculated as the difference between bedtime and rise time. Sleep onset latency (SOL: defined as the length of time that it takes to accomplish the transition from full wakefulness to sleep) and wake after sleep onset (WASO: defined as amount of time a person spends after sleep onset) were also indicated separately for weekdays and weekends in hours and minutes. Sleep duration was defined as TIB minus SOL and WASO.

All participants also indicated the average number of nights per week they experienced difficulties initiating sleep (DIS), difficulties maintaining sleep (DMS), and early morning awakenings (EMA), as well as daytime sleepiness and tiredness. Those suffering from sleep problems were asked about how long the problems had been present. The following 3 criteria were used as an operationalization for insomnia disorder, in line with the DSM-5 criteria: (1) the presence of either DIS, DMS, or EMA for at least 3 nights per week, (2) the presence of daytime sleepiness and tiredness for at least 3 days per week, and (3) a duration of the sleep problems for at least 3 months. More details about the sleep inventory used in SHoT2018 has been published elsewhere (Sivertsen et al., 2019c).

### Alcohol-Related Problems

Potential alcohol-related problems were measured by the Alcohol Use Disorders Identification Test (AUDIT), which is a widely used instrument developed by the World Health Organization to identify risky or harmful alcohol use (Saunders et al., 1993; Babor et al., 2001). The 10-item AUDIT includes items for measuring the frequency, typical amount and episodic heavy drinking frequency (items 1–3), alcohol dependence (items 4–6), and problems related to alcohol consumption (items 7–10) (Shevlin and Smith, 2007). The AUDIT score ranges from 0 to 40. More information about the AUDIT in the SHoT surveys has been published elsewhere (Heradstveit et al., 2019).

### Sociodemographic Information

All participants reported their gender, age and relationship status (coded as single vs. married/partner or girlfriend/boyfriend). Annual income was coded dichotomously according to self-reported income last year (before tax and deductions, and not including loans and scholarships): annual income > 10,000 NOK vs. ≤ 10,000 NOK. Finally, participants were categorized as an immigrant if the student or one or both of the parents were born outside Norway.

### Statistics

IBM SPSS version 27 (SPSS Inc., Chicago, IL USA) for Windows was used for all analyses. Differences between elite athletes and the control group were examined across all continuous outcome measures (HSCL-25, SWLS, T-ILS, EDI-P, EDS-5, PANAS and AUDIT) separately for male and female athletes, by calculating estimated marginal means (EMM), adjusting for age. Differences between elite athletes in team sports and individual sports were also examined using age-adjusted EMM. Cohen's *d* effect-sizes were calculated in line with recognized guidelines (Carlson and Schmidt, 1999; Morris, 2008). As a benchmark for interpreting Cohen's *d*, 0.80 should be regarded as large, 0.50 as moderate, and 0.20 as small, respectively (Cohen, 1988). We also conducted log-link binomial regression analysis to calculate effect-sizes for the dichotomous outcomes (anxiety and depressive disorder, self-harm and suicidal ideation, insomnia and eating disorder), adjusting for age. Results are presented as risk ratios (RR) with 95% confidence intervals. The normality of the data was examined using skewness and kurtosis, and all continuous measures were well within the recommended ranges (+/– 2) (George and Mallery, 2016). *P*-values adjusted for multiple comparisons using the Benjamini-Hochberg's false discovery rate (FDR). There was generally little missing data (*n* < 270 [0.5%]), and hence missing values were handled using listwise deletion. As the SHoT2018 study had several objectives and was not designed to be a study of elite athletes specifically, no *a priori* power calculations were conducted to ensure that the sample size had sufficient statistical power to detect differences in outcomes.

## Results

### Sample Characteristics

In all, 634 of the 50 034 students (1.3%) reported being an elite athlete, of whom 366 (57.8%) were female. The elite athletes were significantly younger than the control group (mean age 22.0 vs. 23.3 years, *P* < 0.001), and the elite athlete group also included a larger proportion of males compared to the control group (42.2 vs. 30.8% [*P* < 0.001], respectively). There were no significant group differences regarding income and ethnicity. More details of the sociodemographic and clinical characteristics among elite athletes vs. non-athletes are found in Table 1.

**Table 1:** Sociodemographic and clinical characteristics of the SHoT 2018 study.

	**Elite athletes**		**Control group**		***p*-value**
**So**	**mean/n**	**SD/%**	**mean/n**	**SD/%**	
Age, mean (SD)	22.03	(2.61)	23.27	(3.30)	<0.001
Gender, *n* (%)					<0.001
Male	366	(57.8%)	34071	(69.2%)	
Female	267	(42.2%)	15132	(30.8%)	
Relationship status, *n* (%)					0.215
Single	302	(47.6%)	24,783	(50.1%)	
Married/partner/girl- or boyfriend	332	(52.4%)	24,637	(49.9%)	
Immigrant status, *n* (%)					0.339
Ethnic Norwegian	590	(93.1%)	45,454	(92.0%)	
Immigrant	44	(6.9%)	3,966	(8.0%)	
Annual income, *n* (%)					0.215
≤ 10,000 NOK	89	(14.0%)	6,187	(12.5%)	
>10,000 NOK	545	(86.0%)	43,233	(87.5%)	
Exercise items, *n* (%)					
Frequency: “*Almost every day”*	634	(100.0%)	49,204	(22.4%)	<0.001
Intensity: “*I push myself to near-exhaustion”*	275	(43.4%)	4,991	(10.7%)	<0.001
Duration: “*More than 1 h”*	582	(92.2%)	16,743	(35.7%)	<0.001
Mental disorder					
Anxiety or depression, % (n)	39	(6.2%)	6,938	(14.0%)	<0.001
Eating disorders, % (n)	12	(1.9%)	1,009	(2.0%)	0.792
Self-harm and suicidal behavior, % (n)	84	(13.2%)	9,231	18.7%)	<0.001
Mental health problems (HSCL-25), mean (SD)	1.52	(0.47)	1.74	(0.55)	<0.001
Life Satisfaction (SWLS), mean (SD)	23.90	(6.39)	21.93	(6.75)	<0.001
Loneliness (T-ILS), mean (SD)	6.65	(2.75)	7.51	(3.07)	<0.001
Perfectionism (EDI-P), mean (SD)	3.27	(0.97)	3.70	(0.99)	<0.001
Disturbed Eating Patterns (EDS-5), mean (SD)	2.63	(1.37)	3.19	(1.41)	<0.001
Positive affect (PANAS), mean (SD)	35.12	(7.56)	30.31	(8.01)	<0.001
Sleep duration, mean (SD)	07:32	(01:31)	07:23	(01:26)	0.01
Insomnia, % (n)	111	(17.5%)	15,167	(30.7%)	<0.001
AUDIT sum score, mean (SD)	5.89	(4.40)	7.27	(4.75)	<0.001

*§ p-values based on overall Chi-squared analyses (categorical variables) or independent samples t-test (continuous variables)*.

### Elite Athletes

**Figure 1:**
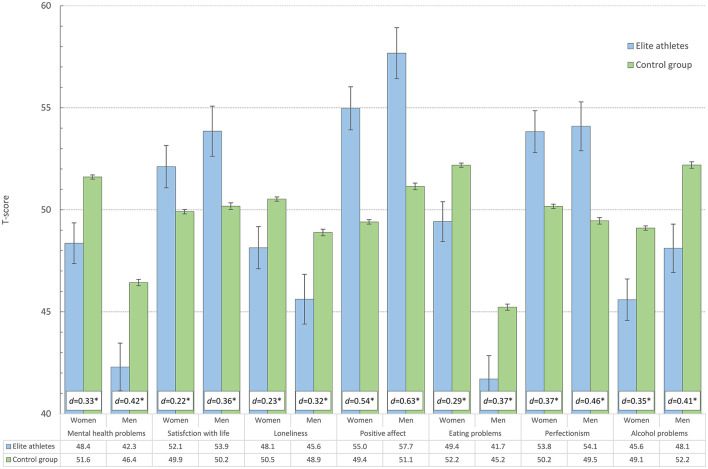
Health indicators among elite athletes and control group in male and female college and university students (age-adjusted estimates represented in T-scores and Cohen's d effect size (in white text box). Error bars represent 95% confidence intervals. *P < 0.001.

**Figure 2:**
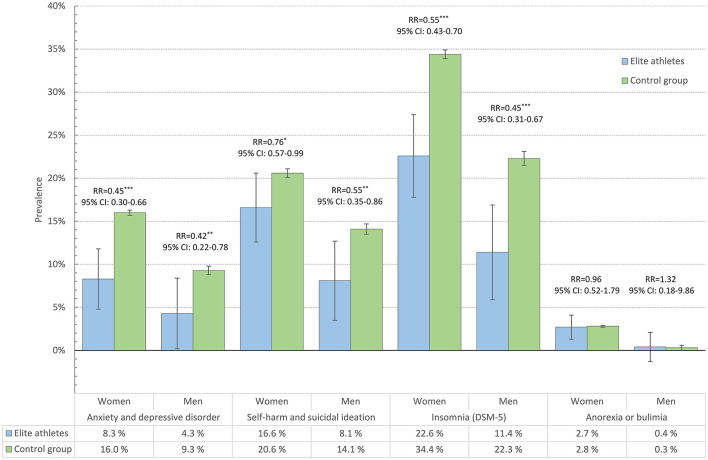
Prevalence of anxiety and depressive disorders, self-harm and suicidal ideation, insomnia and eating disorders among elite athletes and control group, in male and female college and university students. Bars represent age-adjusted prevalence estimates, and error bars represent 95% confidence intervals. RR = age-adjusted relative risk. ***P < 0.001; **P < 0.01; *P < 0.05.

In general, elite athletes reported better mental health across most continuous measures, a trend which was evident in both male and female athletes. As detailed in Figure 1, compared to the control group, elite athletes reported significantly fewer mental health problems (HSCL-25), less loneliness (T-ILS), higher satisfaction with life (SWLS), less disturbed eating patterns (EDS-5), more positive affect (PANAS), as well as fewer alcohol problems (AUDIT). However, elite athletes also reported significantly higher levels of perfectionism compared to the control group. Effect-sizes ranged from small to moderate (Cohen's *d*: 0.2 to 0.6). Similar results were also observed across the dichotomous measures. As displayed in Figure 2, male and female elite athletes had significantly lower prevalence (and significantly lower age-adjusted relative risks) of both anxiety and depressive disorder and self-harm and suicidal ideation, compared to the control group. The prevalence of self-reported anorexia or bulimia did not differ significantly between the two groups. There were no significant sex × group interactions for any of the health indicators.

Elite athletes had a significantly lower prevalence of insomnia than the control group. As displayed in Figure 2, the prevalence of insomnia among male and female elite athletes were 11.4 and 22.6%, respectively, compared to 22.6 and 34.4% among male and female controls. Female elite athletes slept for similar duration to those in the control group (7:26 vs. 7:24 h), whereas male elite athletes slept 12 min longer than the control group (7:35 vs. 7:23 h). However, the sex × group interaction was not statistically significant (*P* = 0.163).

### Individual and Team Sport

Compared to individual sports (*n* = 380), elite athletes in team sports (*n* = 234) had significantly fewer mental health problems (*P* = 0.045), less loneliness, but also more alcohol problems (male athletes only), and more disturbed eating patterns. No differences were observed for quality of life, positive affect, sleep duration or perfectionism.

### Weekly Hours of Exercise

**Figure 3:**
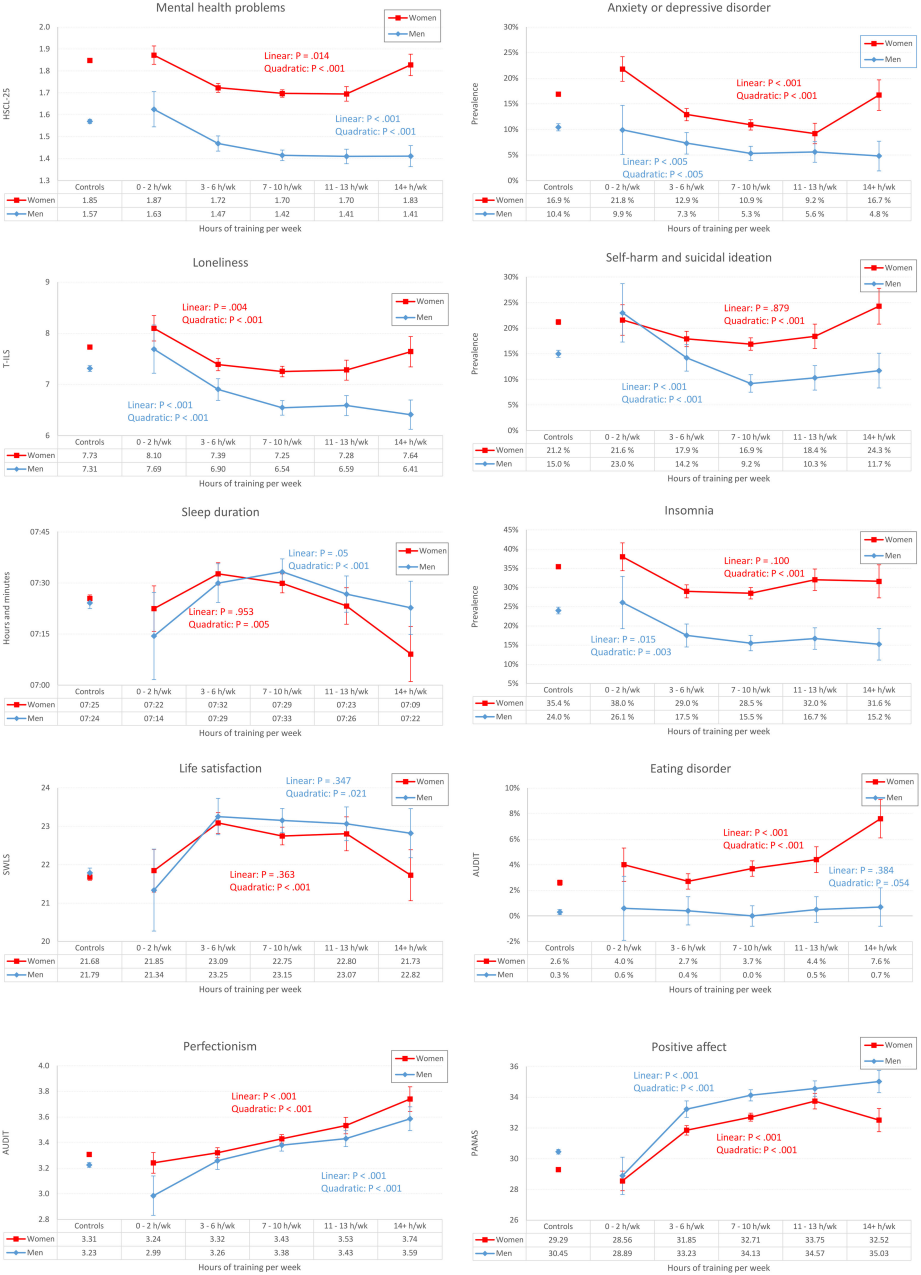
Hours of training (X axis) and age-adjusted health indicators (Y axis) in male and female college and university students exercising “almost every day” (not just elite athletes). The unconnected point estimates (to the left on each panel) represent students training 2–3 days/wk or less. Error bars represent 95% confidence intervals.

The associations between weekly hours of training and all health indicators are detailed in Figure 3. Among male students (blue lines), we observed a positive association across most instruments: the more hours of training, the better the mental health and higher life satisfaction. However, for most outcomes, there was generally little to be gained from increasing the amount of training from 7–10 hours/week to 14+ h per week (Figure 3). Some exceptions should be noted: for alcohol problems we observed a significant curvilinear relationship between hours of training and reported alcohol problems, with the least alcohol problems observed for those training the least *and* the most. In contrast, there were linear relationships throughout all categories of training hours and positive affect: the more training, the more positive affect.

Among female students, we generally found the same pattern of results, but with one gender-specific trend: For female athletes training the most (14+ h per week), there was generally a *worsening* of mental health across most outcome measures. As displayed in Figure 3 (red lines), we found evidence of significant *U-*shaped (curvilinear) associations between training hours and all outcome instruments, except insomnia. The correlations between hours of training per week and mental health problems are detailed in Table 2.

**Table 2:** Pearson correlation coefficient between hours of training and instruments (continuous) assessing mental health problems.

	**Hours of training**
Mental health problems (HSCL-25)	−0.077^**^
Life Satisfaction (SWLS)	0.004
Loneliness (T-ILS)	−0.065^**^
Perfectionism (EDI-P)	−0.120^**^
Disturbed Eating Patterns (EDS-5)	−0.086^**^
Positive affect (PANAS)	0.133^**^
Sleep duration	−0.022^**^
AUDIT sum score	0.047^**^

^*^ p < 0.05;

** *p < 0.01*.

## Discussion

This is the first survey of elite athletes' mental health among students containing a large control group. Both male and female elite athletes had generally better mental health across all examined health outcomes, and elite athletes in team sports had even slightly better mental health compared to athletes in individual sports. The overall pattern was that the more hours of physical exercise, the better the mental health and higher life satisfaction, although there was little to be gained from increasing the amount of training at the extreme end the exercise continuum. Importantly, this study also demonstrated that among female athletes training the most, there was generally a worsening of mental health across most outcome measures.

Despite being crucial for optimal performance, the mental health aspect of elite athletes was historically somewhat neglected for many years, both in the popular media and research literature. One possible reason for this may have been the tendency to idealize elite athletes (Doherty et al., 2016), leading both the general public and health care professionals to assume a low prevalence of mental health issues in sport. Also, athletes may have a negative perception of help-seeking behavior (Steinfeldt and Profile, 2012), and may often minimize any displays of weakness (Sinden, 2010). Similarly, there is also the possibility of stigma which may prevent reporting of a prior diagnosis, which in turn may lead to both underreporting of mental health problems, as well as lack of adequate mental support during their careers (Doherty et al., 2016). Fortunately, recent years have shown a rapid increased in high quality studies in this field, and with several high profile athletes reporting struggling with mental health issues, especially recently during the Tokyo Olympics in 2021 (Park, 2021; Peter, 2021), the times have changed in terms of how we regard the importance of mental health of elite athletes.

Findings from the few existing studies in this field have been mixed, and while a recent systematic review and meta-analyses suggested that the prevalence of mental health problems and disorders in elite athletes might be slightly higher than in the general population, the authors also stressed that the typical lack of control groups limited the generalizability of findings. Also, methodological differences both in how elite athletes are defined, and how mental health is operationalized, may explain some of these inconclusive findings, and there has clearly been a need for large studies with well-defined instruments to further shed light on this important issue. As such, the current study corroborates the findings from one of the largest register studies in this field, examining all US Olympians (*n* = 8124) who participated in the Summer or Winter Games between 1912 and 2012. That study found that elite athletes have significantly *lower* risk of both mental health problems and suicide when compared to the general population (Duncombe et al., 2020). Extending on these findings of reduced risk of mental health problems and suicidality, the current study also found elite athletes reported significantly higher quality of life, more positive affect, less loneliness and insomnia, as well as fewer alcohol problems, when compared to the general student population. As such, when contrasted to the findings from the meta-analyses of Gouttebarge et al. (2019), one may conclude that more well-conducted and well-powered studies are needed to identify possible subgroups of athletes which may be more prone to developing mental health problems. The current study extends on previous research reports by showing that athletes in team sports have slightly better mental health and less loneliness (Elbe and Jensen, 2016; Sabiston et al., 2016; Pluhar et al., 2019), but also somewhat more alcohol problems (males only) (Denault and Poulin, 2018). As such, the mental health benefits of participation in team sports is somewhat nuanced by the increase risk of alcohol problems, when compared to participation in individual sports. However, effect-sizes were generally small, and non-significant for the other outcome measures.

While the current study did not aim to assess potential mechanisms as to why elite athletes may have better mental health than non- athletes, there may be several reasons for these findings. First, we cannot disregard the possibility of selection bias, as optimal mental health is essential when performing at a top international level in any sport. Second, several biological mechanisms have been shown to be involved in the association between physical exercise and mood, and a recent meta-analysis (Morres et al., 2019) showed that extensive physical exercise not only has an anti-depressive effect by boosting endorphin level in the short term, but it may also help to stimulate the functioning of the brain on a broader level. Still, there is clearly a need for both epidemiological and physiological studies that may help shed light on possible involved mechanisms.

A second aim of this study was to examine if there is a linear or curvilinear relationship between hours of exercise and mental health; is more always better? Our general pattern of findings suggests this to be the case, demonstrating that the more hours of physical exercise were associated with better mental health and higher life satisfaction. This is a somewhat different finding than a large study of 1.2 million individuals in the USA. While that study also found a strong link between physical exercise and improved mental health, they also concluded that more exercise was not always better: extreme ranges of more than 23 training sessions per month, or longer than 90 min per session, were associated with worse mental health. This curvilinear association was also partly replicated in the current study, with female (but not male) athletes training the most (14+ h per week), displaying a worsening of mental health across most outcome measures. There may be several reasons for this gender differences. On one hand, the response bias hypothesis suggests that gender differences in mental health may reflect a tendency for men to underreport their problems and symptoms (Sigmon et al., 2005). However, while this may explain the overall gender pattern in this study, this does not fully explain the worsening in mental health among female elite athletes training the most. As such, more studies are needed to further disentangle this important finding. The current findings can also be compared with a US study from 2008 of 7674 adults from the general population, who found a curvilinear association between physical activity and general mental health (Kim et al., 2012). Of interest, that study concluded with an optimal range of 2.5 to 7.5 h of physical activity per week.

However, despite the ongoing discussion regarding the existence of a dose-response relationship between physical exercise and mental health, most will agree that even a small increase in physical exercise from inactivity is beneficial. Although both educational institutions and student welfare organizations try hard to encourage and facilitate their students to take part in a wide range of sports, physical exercise and outdoor activities, the current results suggest that increased efforts are warranted.

### Methodological Considerations

The current study has some important methodological considerations. First, an important limitation of the study is the cross-sectional design, which makes it difficult to evaluate the directionality between physical exercise and mental health. While there is much evidence showing that regular physical exercise has a positive impact on mood (Morres et al., 2019), there are also studies showing that the association is likely to be bidirectional. For example, prospective studies have found that symptoms of depression predict subsequent lower activity levels (Pinto Pereira et al., 2014), and there are also plausible mechanisms which may explain how symptoms of depression may lead to inactivity, including low energy levels or apathy (Goodwin, 2003), psychomotor retardation and anhedonia (Jerstad et al., 2010), and social isolation which in turn may reduce the motivation to be active (Kaplan et al., 1991). Also, our operationalization of “elite athlete” based on self-report among college and university students only, may differ from some other high quality studies, of which some have used more stringent definitions (e.g. competing in the Olympics etc.). Furthermore, the questionnaire we did not include any subcategories of elite athletes, and as such we were unable to consider and compare non-athletes vs. recreational athletes and elite athletes. Similarly, it should be stressed that parallel careers and college/university studies may not be common at the elite level in many large professional sports, which further limits the generalizability of this study. Also, this crude measure did not enable us to explore differences between various sports. In sum, care should be taken when comparing our findings with the existing literature base. Another limitation is the moderate response rate of 31%, and we also had limited information regarding non-responders. Furthermore, due to lack of statistical power, 14+ h per week was the highest category of exercise duration per week. As many world class athletes train 20+ h per week, we were unable to the extreme end of this continuum in detail. The strengths of the study include the large and heterogeneous sample, as most previous studies in this field have examined white, young and female undergraduates (Fedina et al., 2018). Other strengths include well-validated instruments of both physical exercise and mental health outcomes.

## Conclusion

In conclusion, the current study showed that both male and female elite athletes generally had better mental health across a range of health outcomes, when compared to the general student population. The study also found a positive and graded relationship between weekly hours of training and mental health, but also a worsening of mental health for females at the extreme end of exercise continuum.

### What We Already Know?

- Mental health problems seem prevalent among elite athletes, but findings remain inconclusive when compared to the general population.
- Findings are mixed regarding the presence of a linear vs. curvilinear association between weekly hours of training and mental health indicators.

### What Are the New Findings?

- Both male and female elite athletes had generally better mental health across all examined health outcomes.
- Compared to individual sports, athletes in team sports reported better mental health and less loneliness, but also somewhat more alcohol problems (males only).
- The overall pattern was that the more hours of physical exercise, the better the mental health and higher life satisfaction, although there was little to be gained from increasing the amount of training at the extreme end.
- Among female athletes training the most, there was generally a worsening of mental health across most outcome measures.

## Data Availability Statement

The datasets presented in this article are not readily available because of privacy regulations from the Norwegian Regional Committees for Medical and Health Research Ethics (REC). Approval from REC (https://helseforskning.etikkom.no) is a pre-requirement. Guidelines for access to SHoT data are found at https://www.fhi.no/en/more/access-to-data. Requests to access the datasets should be directed to Børge Sivertsen, borge.sivertsen@fhi.no.

## Ethics Statement

All procedures involving human subjects/patients were approved by the Regional Committee for Medical and Health Research Ethics in Western Norway (No. 2017/1176 [SHOT2018]). Electronic informed consent was obtained after the participants had received a detailed introduction to the study.

## Author Contributions

BS contributed with planning and design of the survey data-collection. MG and BS planned, designed, and coordinated the present study. MG and BS conducted the statistical analyses on the survey data, conducted the literature review, and led the writing of the manuscript. BC contributed with input on design and analytical plan, interpretation of results, and critical revision of the manuscript and analyses. All authors approved the submission.

## Funding

SHoT2018 has received funding from the Norwegian Ministry of Education and Research (2017) and the Norwegian Ministry of Health and Care Services (2016).

## Conflict of Interest

The authors declare that the research was conducted in the absence of any commercial or financial relationships that could be construed as a potential conflict of interest.

## Publisher's Note

All claims expressed in this article are solely those of the authors and do not necessarily represent those of their affiliated organizations, or those of the publisher, the editors and the reviewers. Any product that may be evaluated in this article, or claim that may be made by its manufacturer, is not guaranteed or endorsed by the publisher.

## References

[B1] Akesdotter C. Kentta G. Eloranta S. Franck J. (2020). The prevalence of mental health problems in elite athletes. J. Sci. Med. Sport. 23, 329–335. 10.1016/j.jsams.2019.10.022 31806359

[B2] Babor T. F. Higgins-Biddle J. C. Saunders J. B. Monteiro M. G. (2001). AUDIT: The Alcohol Use Disorders Identification Test: Guidelines For Use in Primary Health Care.29627844

[B3] Carlson K. D. Schmidt F. L. (1999). Impact of experimental design on effect size: Findings from the research literature on training. J. Appl. Psychol. 84, 851–862. 10.1037/0021-9010.84.6.851

[B4] Chekroud S. R. Gueorguieva R. Zheutlin A. B. Paulus M. Krumholz H. M. Krystal J. H. (2018). Association between physical exercise and mental health in 1.2 million individuals in the USA between 2011 and 2015: a cross-sectional study. Lancet Psychiat. 5, 739–746. 10.1016/S2215-0366(18)30227-X 30099000

[B5] Cohen J (1988). Statistical Power Analysis for the Behavioral Sicences. 2nd ed. Lawrence Erlbaum Associates.

[B6] Denault A. S. Poulin F. (2018). A detailed examination of the longitudinal associations between individual and team sports and alcohol use. Addict. Behav. 78, 15–21. 10.1016/j.addbeh.2017.10.019 29121528

[B7] Derogatis L. R. Lipman R. S. Rickels K. Uhlenhuth E. H. Covi L. (1974). The Hopkins Symptom Checklist (HSCL): a self-report symptom inventory. Behav. Sci. 19, 1–15. 10.1002/bs.3830190102 4808738

[B8] Diener E. Emmons R. A. Larsen R. J. Griffin S. (1985). The Satisfaction with life scale. J. Person. Assess. 49, 71–75. 10.1207/s15327752jpa4901_13 16367493

[B9] Doherty S. Hannigan B. Campbell M. J. (2016). The experience of depression during the careers of elite male athletes. Front. Psychol. 7, 1069. 10.3389/fpsyg.2016.01069 27486418 PMC4947597

[B10] Duncombe S. L. Tanaka H. De Larochelambert Q. Schipman J. Toussaint J. F. Antero J. (2020). High hopes: lower risk of death due to mental disorders and self-harm in a century-long US Olympian cohort compared with the general population. Br. J. Sports. 55, 900–905. 10.1136/bjsports-2020-102624 33214139

[B11] Eijsvogels T. M. H. Thompson P. D. (2015). Exercise is medicine. At any dose? JAMA. 314, 1915–1916. 10.1001/jama.2015.10858 26547459

[B12] Elbe A. M. Jensen S. N. (2016). Commentary: comparison of athletes' proneness to depressive symptoms in individual and team sports: research on psychological mediators in junior elite athletes. Front. Psychol. 7. 10.3389/fpsyg.2016.01782 27917134 PMC5114271

[B13] Fedina L. Holmes J. L. Backes B. L. (2018). Campus sexual assault: a systematic review of prevalence research from 2000 to 2015. Trauma. Violence Abus. 19, 76–93. 10.1177/1524838016631129 26906086

[B14] Garner D. Olmsted M. Polivy J. (1985). Eating disorder inventory. Psychopharmacol. Bull. 21, 1009–1010.

[B15] George D. Mallery P. (2016). IBM SPSS Statistics 23 Step by Step: A Simple Guide and Reference. 13th ed. New York: Routledge. 10.4324/9781315545899

[B16] Glaesmer H. Braehler E. Grande G. Hinz A. Petermann F. Romppel M. (2014). The German Version of the Hopkins Symptoms Checklist-25 (HSCL-25)—factorial structure, psychometric properties, and population-based norms. Comput. Psychiat. 55, 396–403. 10.1016/j.comppsych.2013.08.020 24286991

[B17] Goodwin R. D (2003). Association between physical activity and mental disorders among adults in the United States. Prev. Med. 36, 698–703. 10.1016/S0091-7435(03)00042-2 12744913

[B18] Gordon B. R. McDowell C. P. Hallgren M. Meyer J. D. Lyons M. Herring M. P. (2018). Association of efficacy of resistance exercise training with depressive symptoms meta-analysis and meta-regression analysis of randomized clinical trials. JAMA Psychiat. 75, 566–576. 10.1001/jamapsychiatry.2018.0572 29800984 PMC6137526

[B19] Gouttebarge V. Castaldelli-Maia J. M. Gorczynski P. Hainline B. Hitchcock M. E. Kerkhoffs G. M. (2019). Occurrence of mental health symptoms and disorders in current and former elite athletes: a systematic review and meta-analysis. Br. J. Sports Med. 53, 700–706. 10.1136/bjsports-2019-100671 31097451 PMC6579497

[B20] Grasdalsmoen M. Engdahl B. Fjeld M. K. Steingrimsdottir O. A. Nielsen C. S. Eriksen H. R. (2020b). Physical exercise and chronic pain in university students. PLoS ONE. 15, e0235419. 10.1371/journal.pone.0235419 32589694 PMC7319292

[B21] Grasdalsmoen M. Eriksen H. R. Lonning K. J. Sivertsen B. (2019). Physical exercise and body-mass index in young adults: a national survey of Norwegian university students. BMC Public Health. 19, 1354. 10.1186/s12889-019-7650-z 31646998 PMC6813074

[B22] Grasdalsmoen M. Eriksen H. R. Lonning K. J. Sivertsen B. (2020a). Physical exercise, mental health problems, and suicide attempts in university students. BMC Psychiat. 20, 175. 10.1186/s12888-020-02583-3 32299418 PMC7164166

[B23] Heradstveit O. Skogen J. C. Brunborg G. S. Lonning K. J. Sivertsen B. (2019). Alcohol-related problems among college and university students in Norway: extent of the problem. Scand. J. Public Health. 1403494819863515. 10.1177/1403494819863515 31319770

[B24] Hughes M. E. Waite L. J. Hawkley L. C. Cacioppo J. T. (2004). A short scale for measuring loneliness in large surveys: Results from two population-based studies. Res. Aging. 26, 655–672. 10.1177/0164027504268574 18504506 PMC2394670

[B25] Jerstad S. J. Boutelle K. N. Ness K. K. Stice E. (2010). Prospective reciprocal relations between physical activity and depression in female adolescents. J. Consult Clin. Psychol. 78, 268–272. 10.1037/a0018793 20350037 PMC2847789

[B26] Kaplan G. A. Lazarus N. B. Cohen R. D. Leu D. J. (1991). Psychosocial factors in the natural history of physical activity. Am. J. Prev. Med. 7, 12–17. 10.1016/S0749-3797(18)30959-0 1867895

[B27] Kim Y. S. Park Y. S. Allegrante J. P. Marks R. Ok H. Cho K. O. (2012). Relationship between physical activity and general mental health. Prev. Med. 55, 458–463. 10.1016/j.ypmed.2012.08.021 22981733

[B28] Knapstad M. Sivertsen B. Knudsen A. K. Smith O. R. F. Aaro L. E. Lonning K. J. (2019). Trends in self-reported psychological distress among college and university students from 2010 to 2018. Psychol. Med. 51, 470–478. 10.1017/S0033291719003350 31779729 PMC7958482

[B29] Krokstad S. Langhammer A. Hveem K. Holmen T. L. Midthjell K. Stene T. R. (2013). Cohort Profile: the HUNT Study, Norway. Int. J. Epidemiol. 42, 968–977. 10.1093/ije/dys095 22879362

[B30] Kurtze N. Rangul V. Hustvedt B. E. Flanders W. D. (2007). Reliability and validity of self-reported physical activity in the Nord-Trondelag Health Study (HUNT 2). Eur. J. Epidemiol. 22, 379–387. 10.1007/s10654-007-9110-9 17356925

[B31] Kurtze N. Rangul V. Hustvedt B. E. Flanders W. D. (2008). Reliability and validity of self-reported physical activity in the Nord-Trondelag Health Study: HUNT 1. Scand. J. Public Health. 36, 52–61. 10.1177/1403494807085373 18426785

[B32] Kvam S. Kleppe C. L. Nordhus I. H. Hovland A. (2016). Exercise as a treatment for depression: a meta-analysis. J. Affect. Disorders. 202, 67–86. 10.1016/j.jad.2016.03.063 27253219

[B33] Lampard A. M. Byrne S. M. McLean N. Fursland A. (2012). The Eating Disorder Inventory-2 Perfectionism scale: factor structure and associations with dietary restraint and weight and shape concern in eating disorders. Eat. Behav. 13, 49–53. 10.1016/j.eatbeh.2011.09.007 22177396

[B34] Lee I. M. Shiroma E. J. Lobelo F. Puska P. Blair S. N. Katzmarzyk P. T. (2012). Effect of physical inactivity on major non-communicable diseases worldwide: an analysis of burden of disease and life expectancy. Lancet. 380, 219–229. 10.1016/S0140-6736(12)61031-9 22818936 PMC3645500

[B35] Lehman E. J. Hein M. J. Gersic C. M. (2016). Suicide Mortality Among Retired National Football League Players Who Played 5 or More Seasons. Am. J. Sports Med. 44, 2486–2491. 10.1177/0363546516645093 27159317 PMC5048489

[B36] Maron B. J. Haas T. S. Murphy C. J. Ahluwalia A. Rutten-Ramos S. (2014). Incidence and causes of sudden death in U.S. college athletes. J. Am. Coll. Cardiol. 63, 1636–1643. 10.1016/j.jacc.2014.01.041 24583295

[B37] Matthews-Ewald M. R. Zullig K. J. (2013). Evaluating the performance of a short loneliness scale among college students. J. Coll. Student Dev. 54, 105–109. 10.1353/csd.2013.0003

[B38] McManus S. Bebbington P. Jenkins R. Brugha T. (2016). Mental Health and Wellbeing in England: Adult Psychiatric Morbidity Survey 2014. Leeds: NHS Digital.

[B39] Mons U. Hahmann H. Brenner H. (2014). A reverse J-shaped association of leisure time physical activity with prognosis in patients with stable coronary heart disease: evidence from a large cohort with repeated measurements. Heart. 100, 1043–1049. 10.1136/heartjnl-2013-305242 24829374

[B40] Morres I. D. Hatzigeorgiadis A. Stathi A. Comoutos N. Arpin-Cribbie C. Krommidas C. (2019). Aerobic exercise for adult patients with major depressive disorder in mental health services: a systematic review and meta-analysis. Depress Anxiety. 36, 39–53. 10.1002/da.22842 30334597

[B41] Morris S. B (2008). Estimating effect sizes from pretest-posttest-control group designs. Organ Res Methods. 11, 364–386. 10.1177/109442810629105919271847

[B42] Muro-Sans P. Amador-Campos J. A. Pero-Cebollero M. (2006). Factor structure of Eating Disorders Inventory-2 in a Spanish sample. Eat. Weight Disord. 11, e42–52. 10.1007/BF03327759 16809969

[B43] Park A (2021). How the Tokyo Olympics Changed the Conversation About Athletes' Mental Health. Time.

[B44] Peter J (2021). Will Tokyo Olympics be a Game-Changer in How We View Athletes' Mental Health? USA Today.

[B45] Pinto Pereira S. M. Geoffroy M. C. Power C. (2014). Depressive symptoms and physical activity during 3 decades in adult life: bidirectional associations in a prospective cohort study. JAMA Psychiat. 71, 1373–1380. 10.1001/jamapsychiatry.2014.1240 25321867

[B46] Pluhar E. McCracken C. Griffith K. L. Christino M. A. Sugimoto D. Meehan W. P. (2019). Team sport athletes may be less likely to suffer anxiety or depression than individual sport athletes. J. Sport Sci. Med. 18, 490–6. 31427871 PMC6683619

[B47] Purcell R. Rice S. Butterworth M. Clements M. (2020). Rates and correlates of mental health symptoms in currently competing elite athletes from the Australian national high-performance sports system. Sports Med. 50, 1683–1694. 10.1007/s40279-020-01266-z 32026315

[B48] Rao A. L. Asif I. M. Drezner J. A. Toresdahl B. G. Harmon K. G. (2015). Suicide in national collegiate athletic association (NCAA) athletes: a 9-year analysis of the NCAA resolutions database. Sports Health. 7, 452–457. 10.1177/1941738115587675 26502423 PMC4547116

[B49] Reardon C. L. Factor R. M. (2010). Sport psychiatry: a systematic review of diagnosis and medical treatment of mental illness in athletes. Sports Med. 40, 961–980. 10.2165/11536580-000000000-00000 20942511

[B50] Reardon C. L. Hainline B. Aron C. M. Baron D. Baum A. L. Bindra A. (2019). Mental health in elite athletes: International Olympic Committee consensus statement (2019). Br. J. Sports Med. 53, 667–699. 10.1136/bjsports-2019-100715 31097450

[B51] Rice S. M. Purcell R. de Silva S. Mawren D. McGorry P. D. Parker A. G. (2016). The mental health of elite athletes: a narrative systematic review. Sports Med. 46, 1333–1353. 10.1007/s40279-016-0492-2 26896951 PMC4996886

[B52] Rosenvinge J. H. Perry J. A. Bjorgum L. Bergersen T. D. Silvera D. H. Holte A. (2001). A new instrument measuring disturbed eating patterns in community populations: development and initial validation of a five-item scale (EDS-5). Eur. Eat. Disord. Rev. 9, 123–132. 10.1002/erv.37125855820

[B53] Sabiston C. M. Jewett R. Ashdown-Franks G. Belanger M. Brunet J. O'Loughlin E. (2016). Number of years of team and individual sport participation during adolescence and depressive symptoms in early adulthood. J. Sport Exer. Psy. 38, 105–110. 10.1123/jsep.2015-0175 27018562

[B54] Saunders J. B. Aasland O. G. Babor T. F. De la Fuente J. R. Grant M. (1993). Development of the alcohol use disorders identification test (AUDIT): WHO collaborative project on early detection of persons with harmful alcohol consumption-II. Addiction. 88, 791–804. 10.1111/j.1360-0443.1993.tb02093.x 8329970

[B55] Schaal K. Tafflet M. Nassif H. Thibault V. Pichard C. Alcotte M. (2011). Psychological balance in high level athletes: gender-based differences and sport-specific patterns. PLoS ONE. 6, e19007. 10.1371/journal.pone.0019007 21573222 PMC3087722

[B56] Schnohr P. O'Keefe J. H. Marott J. L. Lange P. Jensen G. B. (2015). Dose of jogging and long-term mortality: the Copenhagen City Heart Study. J. Am. Coll. Cardiol. 65, 411–419. 10.1016/j.jacc.2014.11.023 25660917

[B57] Shevlin M. Smith G. W. (2007). The factor structure and concurrent validity of the alcohol use disorder identification test based on a nationally representative UK sample. Alcohol. Alcoholism. 42, 582–587. 10.1093/alcalc/agm045 17660524

[B58] Sigmon S. T. Pells J. J. Boulard N. E. Whitcomb-Smith S. Edenfield T. M. Hermann B. A. (2005). Gender differences in self-reports of depression: the response bias hypothesis revisited. Sex Roles. 53, 401–411. 10.1007/s11199-005-6762-3

[B59] Sinden J. L (2010). The normalization of emotion and the disregard of health problems in elite amateur sport. J. Clin. Sport Psychol. 4, 241–256. 10.1123/jcsp.4.3.241

[B60] Sivertsen B. Hysing M. Knapstad M. Harvey A. G. Reneflot A. Lonning K. J. (2019b). Suicide attempts and non-suicidal self-harm among university students: prevalence study. Bjpsych Open. 5. 10.1192/bjo.2019.4 31068238 PMC6401540

[B61] Sivertsen B. Rakil H. Munkvik E. Lonning K. J. (2019a). Cohort profile: the SHoT-study, a national health and well-being survey of Norwegian university students. BMJ Open. 9, e025200. 10.1136/bmjopen-2018-025200 30670525 PMC6347864

[B62] Sivertsen B. Vedaa O. Harvey A. G. Glozier N. Pallesen S. Aaro L. E. (2019c). Sleep patterns and insomnia in young adults: A national survey of Norwegian university students. J. Sleep. Res. 28, e12790. 10.1111/jsr.12790 30515935

[B63] Skogen J. C. Øverland S. Smith O. R. Aarø L. E. (2017). The factor structure of the Hopkins Symptoms Checklist (HSCL-25) in a student population: a cautionary tale. Scand. J. Public Health. 45, 357–365. 10.1177/1403494817700287 28381118

[B64] Steinfeldt J. A. S. Profile M. C. (2012). of masculine norms and help -seeking stigma in college football. Sport Exer. Performan. Psychol. 1, 58–71. 10.1037/a0024919

[B65] Ventevogel P. De Vries G. Scholte W. F. Shinwari N. R. Faiz H. Nassery R.. (2007). Properties of the Hopkins Symptom Checklist-25 (HSCL-25) and the Self-Reporting Questionnaire (SRQ-20) as screening instruments used in primary care in Afghanistan. Soc. Psychiat. Psychiatr. Epidemiol. 42, 328–335. 10.1007/s00127-007-0161-8 17370049

[B66] Williams P. T. Thompson P. D. (2014). Increased cardiovascular disease mortality associated with excessive exercise in heart attack survivors. Mayo. Clin. Proc. 89, 1187–1194. 10.1016/j.mayocp.2014.05.006 25128072

